# Assessment of Psyllid Handling and DNA Extraction Methods in the Detection of ‘*Candidatus* Liberibacter Solanacearum’ by qPCR

**DOI:** 10.3390/microorganisms10061104

**Published:** 2022-05-26

**Authors:** María Quintana, Leandro de-León, Jaime Cubero, Felipe Siverio

**Affiliations:** 1 Unidad de Protección Vegetal, Instituto Canario de Investigaciones Agrarias, 38270 San Cristóbal de La Laguna, Spain; fsiverio@icia.es; 2 Departamento de Bioquímica, Microbiología, Biología Celular y Genética, Facultad de Farmacia, Universidad de La Laguna, 38200 San Cristóbal de La Laguna, Spain; lleongue@ull.es; 3 Centro Nacional Instituto de Investigación y Tecnología Agraria y Alimentaria (INIA/CSIC), 28040 Madrid, Spain; cubero@inia.es; 4 Sección de Laboratorio de Sanidad Vegetal, Consejería de Agricultura, Ganadería, Pesca y Aguas del Gobierno de Canarias, 38270 San Cristóbal de La Laguna, Spain

**Keywords:** vector, disease, bacterium, *Bactericera trigonica*, HLB, ethanol contamination

## Abstract

‘*Candidatus* Liberibacter solanacearum’ (CaLsol) is an uncultured bacterium, transmitted by psyllids and associated with several diseases in *Solanaceae* and *Apiaceae* crops. CaLsol detection in psyllids often requires insect destruction, preventing a subsequent morphological identification. In this work, we have assessed the influence on the detection of CaLsol by PCR in *Bactericera trigonica* (Hemiptera: Psyllidae), of four specimen preparations (entire body, ground, cut-off head, and punctured abdomen) and seven DNA extraction methods (PBS suspension, squashing on membrane, CTAB, Chelex, TRIsure^TM^, HotSHOT, and DNeasy^®^). DNA yield and purity ratios, time consumption, cost, and residues generated were also evaluated. Optimum results were obtained through grinding, but it is suggested that destructive procedures are not essential in order to detect CaLsol. Although CaLsol was detected by qPCR with DNA obtained by the different procedures, HotSHOT was the most sensitive method. In terms of time consumption and cost, squashed on membrane, HotSHOT, and PBS were the fastest, while HotSHOT and PBS were the cheapest. In summary, HotSHOT was accurate, fast, simple, and sufficiently sensitive to detect this bacterium within the vector. Additionally, cross-contamination with CaLsol was assessed in the ethanol solutions where *B. trigonica* specimens were usually collected and preserved. CaLsol-free psyllids were CaLsol-positive after incubation with CaLsol-positive specimens. This work provides a valuable guide when choosing a method to detect CaLsol in vectors according to the purpose of the study.

## 1. Introduction

‘*Candidatus* Liberibacter solanacearum’ (CaLsol) is an uncultured, phloem-limited, Gram-negative bacterium of the alphaproteobacteria class belonging to the Rhizobiaceae family [1]. It is associated with Zebra Chip in potato and several vegetative disorders in *Solanaceae* and *Apiaceae* crops [2,3,4,5,6,7]. Eleven haplotypes of CaLsol were described according to their geographical distributions, host plant, and insect vector [7,8,9,10,11,12]. Haplotypes A and B are considered as regulated non-quarantine pests in the European legislation (Commission implementing regulation-EU 2019/2072). These haplotypes have been associated with Zebra Chip in solanaceous in North and Central America, whereas haplotype A is also found in New Zealand [13]. Haplotype C is present in the North of Europe [8,9,14,15,16] and haplotypes D and E were described in southern Europe, northern Africa, and in the Mediterranean basin [9,17,18,19,20,21,22,23]. The last three haplotypes are mainly detected in *Apiaceae*, but CaLsol C and CaLsol E were also found in potatoes in Finland and Spain, respectively [24,25,26]. Haplotype U has been described in *Urtica dioica* L. [10], haplotype F in a single potato tuber in the United States [11] and haplotype G was found in *Solanum umbelliferum* Eschsch. in California (USA) [27]. Two haplotypes designated as H were described, one in carrots and in two species of *Poligonaceae*: *Persicaria lapathifolia* L. and *Fallopia convolvulus* L. in Finland [12], and another in *Convolvulaceae* species in USA [28]. Finally, haplotypes Cra1 and Cra2 have been found in psyllids from the Aphalaridae family: *Craspedolepta nebulosa* (Zetterstedt, 1828) and *C. subpunctata* (Foerster, 1848) in United Kingdom [29].

Currently, six species of psyllids (Hemiptera: Triozidae) have been described as CaLsol vectors. *Bactericera trigonica* Hodkinson, 1981 lives in carrot-producing areas of southern Europe, northern Africa and the Near East, next to the Mediterranean basin and the nearby Atlantic Coast [30,31,32]. This species feeds primarily in carrot crops, but it can also be found in other species of *Apiaceae* transmitting CaLsol [7,18]. The potato psyllid, *B. cockerelli* (Šulc, 1909), which naturally affects potato and tomato, is the cited vector for haplotypes A and B [33]. *Bactericera nigricornis* Foerster, 1848, which naturally affects carrot and potato, may transmit haplotypes D and E [34,35]. *Trioza apicalis* Foerster, 1948, naturally affects carrots and was associated to haplotype C [8]. Finally, the psyllids *T. urticae* (Linné, 1758) and *T. anthrisci* Burckhardt, 1986, are vectors of haplotype U [10,36].

International trade of plants, vegetables, and fruits between different countries has contributed to the worldwide spread of harmful pests. The correct taxonomic identification of the pest is crucial in order to adopt the most appropriate and effective control measures. This identification may be achieved by classical morphological identification or by DNA-based molecular approaches. Usually, DNA extraction methods from arthropod samples involve the destruction of the specimen, preventing its subsequent morphological identification [37,38,39,40,41,42,43,44]. Although the preservation of the whole specimen structure is not always considered, some researchers have approached the use of non-destructive DNA extraction techniques to obtain both morphological and molecular identification [45,46,47,48,49]. Comparative studies of different DNA extraction methods have been performed using commercial extraction kits that offer standardized methods to ensure reliable results [39,41,50]. However, these kits are often expensive and their use becomes impractical to process a large number of samples or when funding resources are limited.

Insects not only produce direct harm to plants when they feed, but also indirect harm by transmitting diseases into the crops. Diseases transmitted by insects cause great economic losses in production areas and are one of the main concerns of the sector. Thus, in some cases, analysis of insect specimens for the detection of vectored plant pathogen is a first step to study the involvement of the insect in disease transmission. DNA extraction procedures used for the molecular identification of arthropods might not be appropriate to detect the pathogen they carry. Besides, the carried microorganism is generally unevenly distributed or at low concentrations in insect tissues and therefore not easily detected, as in the case of CaLsol [43].

In addition, water traps with detergents and preservatives—such as polyethylene glycol—are used for insect capture. The insects are usually collected and preserved altogether in ethanol solutions supplemented with glycerol. The use of these solutions to capture or preserve insect vectors could provide a source of cross-contamination with the target microorganism. This might lead to incorrect interpretation in studies of CaLsol prevalence in insect populations.

The overall objective of this work was to provide an appropriate method to detect CaLsol in its vectors, allowing their subsequent identification. Four specimen preparations and seven DNA extraction methods were assessed and compared in terms of DNA yield and purity ratio, results of conventional and real-time PCR (qPCR) analysis, time consumption, cost, and residues generated. Additionally, cross-contamination with CaLsol was also evaluated in the ethanol solutions where *B. trigonica* specimens are usually collected and preserved.

## 2. Materials and Methods

### 2.1. Source of Insects

Psyllids were collected from an experimental carrot field in the Instituto Canario de Investigaciones Agrarias (ICIA), located in Valle Guerra (Tenerife, Spain). They were captured using an entomological net and immediately taken to the laboratory for identification using a binocular microscope. *Bactericera trigonica* specimens were confined in entomological cages and fed on pesticide-free carrot plants (*Daucus carota* L. var. Bangor F1) infected with CaLsol to establish a positive colony. Under these conditions, psyllids were maintained for more than three months to ensure the acquisition of the bacteria. The CaLsol-free specimens were provided by the Instituto de Ciencias Agrarias of Consejo Superior de Investigaciones Científicas from Madrid (ICA-CSIC, Madrid, Spain). To guarantee the presence and absence of CaLsol in positive and negative colonies, respectively, adults from these colonies were randomly tested with two validated qPCR protocols: the CaLsol protocol [7] and the Lso protocol [51].

Specimens were collected from the colonies and killed by freezing at −20 °C without aqueous solution. The insects were then identified and sexed using a binocular microscope and processed individually according to each assay as described in subsequent sections.

### 2.2. Insect Preparations

All DNA extraction methods were evaluated on ground material. Each specimen was manually ground with a tapered pestle in 1.5 mL microtube with 5 µL of absolute ethanol. Subsequently, the ethanol was evaporated at room temperature for 15 min.

Three additional non-destructive preparations were also evaluated: (i) intact full insects without treatment or manipulation (hereinafter ‘whole’); (ii) decapitated insects by splitting the head from the rest of the body (‘cut off head’); and (iii) abdomen punctured once by using a sterile entomological needle (‘punctured abdomen’).

### 2.3. DNA Extraction Methods

The number and type of samples used in each DNA extraction method and specimen preparation are summarized in Table 1. Seven DNA extraction procedures were assessed: CTAB [52], Chelex (Bio-Rad Laboratories, Hercules, CA, USA) [53], TRIsure^TM^ (Bioline, London, UK), squashed on membrane [54], HotSHOT [55,56], PBS suspension, and DNeasy Blood and Tissue kit^®^ (QIAGEN, Hilden, Germany) (hereinafter ‘DNeasy^®^’) [49]. Fourteen individuals of *B. trigonica* (seven females and seven males) were used to evaluate each combination specimen preparation/method. All DNA extraction methods were assessed and compared from ground specimen preparation. In addition, the three non-destructive preparations were evaluated using Chelex, HotSHOT, PBS, and DNeasy^®^ (14 specimens per method and preparation). Description and modifications of the DNA extraction methods are explained below. Extracted DNA was stored at −20 °C until use.

CTAB method described by Murray and Thompson [52] was carried out with some modifications. First, 400 µL of CTAB buffer (2% (weight/volume, *w*/*v*) CTAB in 100 mM Tris HCl 1 M, 1.4 M NaCl, 20 mM EDTA 0.5 M, 1% (*w*/*v*) PVP, 0.1% (*w*/*v*) sodium bisulphite) were added to the ground specimen and heated at 65 °C for 15 min. The microtube was manually shaken, heated again at 65 °C for 15 min, and centrifuged at 850 rcf for 5 min. Supernatant (400 µL) was collected and carefully mixed with an equal volume of chloroform: isoamyl alcohol (24:1) and centrifuged at 18,500 rcf for 5 min. Two hundred microliters of the supernatant were mixed with 120 µL of isopropanol and kept at −20 °C for 30 min. The mixture was centrifuged at 18,500 rcf for 20 min, and the supernatant was discarded before the addition of 1 mL of 70% ethanol. The samples were mixed and centrifuged at 18,500 rcf for 10 min and the supernatant discarded, without disturbing the pellet. Finally, the pellet was dried for 2 h at room temperature and resuspended in 100 µL of RNAse-DNAse free water.

Chelex 100 (Biorad) was performed according to Casquet et al. [53]. Ten µL of proteinase k (10 mg·mL^−1^) and 150 µL of Chelex (10% Chelex 100 Resin, 0.1 mg·mL^−1^) were added to each sample, incubated at 55 °C for 24 h, and then cooled to room temperature.

TRIsure^TM^ (Bioline, Sydney, Australia) method was performed according to the manufacturer’s instruction [57]: 1.11 mL of TRIsure^TM^ were added to the specimen preparation, mixed with a vortex and centrifuged at 12,000 rcf for 7 min. The supernatant was collected, mixed with 200 µL of chloroform, and shaken for 10–15 s before centrifugation at 12,000 rcf for 15 min. The aqueous phase was removed and 350 µL of absolute ethanol were added to organic phase, homogenized by mixing and centrifuged at 12,000 rcf for 7 min. The supernatant was discarded and the pellet was washed twice for 2 min with 1 mL of 0.1 M sodium citrate supplemented with 10% ethanol before centrifugation at 12,000 rcf for 7 min. The pellet was washed once with 1.5 mL of 75% ethanol, gently mixed for 30 s and centrifuged at 12,000 rcf for 7 min. The supernatant was discarded and the resultant pellet was dried out at 65 °C for 2 h. Finally, the pellet was resuspended in 50 μL of RNAse-DNAse free water.

For squashed on membrane method [54], a specimen was crushed with the bottom of a microtube on a positively charged nylon membrane (NYLM-Ro Roche, Merck KGaA, Darmstadt, Germany, ref. 11209299001). The piece of membrane with the squashed insect (~0.5 cm^2^) was introduced to a microtube with 100 µL of RNAse–DNAse free water. The sample was vortexed and incubated at room temperature for 5 min before use.

For HotSHOT method [55,56], each specimen was heated at 100 °C for 15 min in 20 µL of 25 mM NaOH (pH = 12). The sample was cooled at 4 °C for 5 min and 20 µL of 40 mM Tris-HCl (pH = 5) was added to neutralize the reaction, at which point the sample was ready for analysis.

For PBS method, 100 µL of buffer (8.0 g NaCl, 2.4 g Na_2_HPO_4_·12H_2_O, 0.2 g KH_2_PO_4_, 0.2 g KCl, 1 L H_2_O, pH 7.2–7.4) supplemented with Tween 20 (0.05%) was added to the specimen preparation, mixed with an orbital shaker for 30 min, and pulse centrifuged.

For DNeasy^®^ method, the commercial DNA extraction kit Blood and Tissue kit^®^ from Qiagen was used according to Sjölund [49].

### 2.4. DNA Yield and Purity

Quantity (ng·μL^−1^) and quality (A_260_/A_280_) of extracted DNA were measured with spectrophotometer ND100 (NanoDrop Technologies, Wilmington, DE, USA). To evaluate the efficiency of each extraction procedure, the DNA yield ratio was calculated considering the resuspension volume and body weight of the psyllid [41] according to the Formula (1):

(1) $$\mathrm{DNA}\;\mathrm{yield}\;\mathrm{ratio}\;(\mathrm{ng}\cdot {\mathrm{mg}}^{-1})=\mathrm{DNA}\;\mathrm{quantity}\;(\mathrm{ng}\cdot \mu L)/\mathrm{volume}\;\mathrm{of}\;\mathrm{DNA}\;\mathrm{resuspension}\;(\mu L)\;\mathrm{mean}\;B.\;trigonica\;\mathrm{body}\;\mathrm{weight}\;\mathrm{ng}$$

Groups of 31 males and 18 females of *B. trigonica* were weighed, obtaining 9.6 mg (an average of 309 ng/specimen) and 8.3 mg (461 ng/specimen), respectively.

### 2.5. Amplification Conditions by Conventional and qPCR

Conventional PCR was used to detect CaLsol by using Lso TX 16/23 primers [58] and KAPA3G Plant PCR Kit (2×) (KAPA Biosystems, Cape Town, South Africa). PCRs were carried out using a SimpliAmp Thermal Cycler (Applied Biosystems, Forest City, CA, USA). The amplification products were visualized in 1.5% (*w*/*v*) agarose gel in 0.5 M TAE buffer (40 mM Tris base, 40 mM acetic acid, 1 mM EDTA) and stained with Good View^TM^ (SBS Genentech Co., Ltd., Beijing, China). Gel images were recorded and processed with EL Logic 100 Image system and Kodak Molecular Imaging software v.4.0.5.

The qPCR analyses were performed in StepOne Plus thermal cycler (Applied Biosystems) with SensiFAST Probe Hi-ROX kit (Bioline) following two protocols previously mentioned [7,51]. The fluorescence threshold was established automatically by the software based on the background noise for the plate. The qPCR detections were repeated with the samples preserved at −80 °C for 130 weeks, to evaluate the storing effect on the results.

Table S1 (Supplementary Materials), shows the sequences and amplification conditions of the PCR and qPCR protocols used in the present study. 

### 2.6. Morphological State of Psyllids after DNA Extraction

Specimens exposed to non-destructive treatments for DNA extraction (DNeasy^®^, PBS, HotSHOT and Chelex) were examined with a stereomicroscope (Nikon SMZ800, Tokyo, Japan). The psyllid body parts with taxonomic characters (forewings, head, metathoracic legs, male and female terminalia, etc.) of treated and non-treated psyllids were compared.

### 2.7. Time Consumed, Cost Estimation, and Residues Generated

The time to complete each DNA extraction method was estimated with a group of 10 samples that were processed at the same time, from the first step until the DNA was ready for PCR reaction. The generation of hazardous waste during DNA extraction was also recorded for the same 10 samples. The cost per sample for the entire process was estimated for each extraction method based on the current prices of consumables and reagents at the time of the evaluation.

### 2.8. Cross-Contamination Assays

To evaluate if cross-contamination occurs during capture and handling of psyllids, three different assays were performed with *B. trigonica*. For all assays, tubes with CaLsol-free and CaLsol-positive psyllids were used as negative and positive controls, respectively.

#### 2.8.1. Assay 1

**Figure 1:**
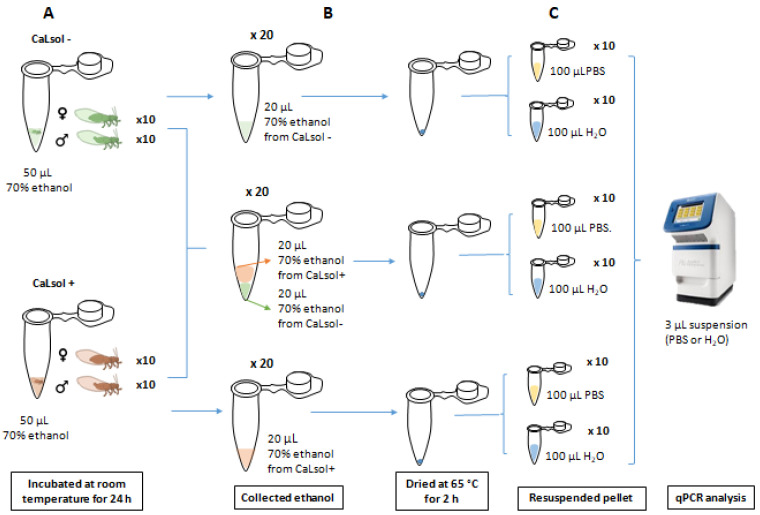
Cross-contamination study: Assay 1. CaLsol-positive and CaLsol-negative psyllids were individually incubated in Eppendorf tubes with 70% ethanol (**A**). Aliquots of ethanol were individually transferred to tubes or by mixing aliquots of positive and negative samples prior to drying (**B**). The samples were resuspended in water or PBS before direct qPCR analysis (**C**). ♀: female; ♂: male.

Twenty CaLsol-positive psyllids (10 males and 10 females), and 20 CaLsol-free psyllids (10 males and 10 females), were introduced in a microtube (one specimen per tube) with 50 μL of 70% ethanol and incubated at room temperature for 24 h (Figure 1). Next, 20 μL of ethanol from tubes with CaLsol-positive insects were taken and mixed with 20 μL of ethanol from tubes containing CaLsol-free specimens. Additionally, volumes of ethanol (20 μL) from tubes with positive and negative specimens were also individually analyzed. The ethanol of the tubes was dried at 65 °C for 2 h. Half of the tubes were then resuspended in 100 μL of RNAse-DNAse free H_2_O and the other half in 100 μL of PBS. Volumes of 3 μL from H_2_O and PBS solutions were used as a template for qPCR following Lso protocol [51].

#### 2.8.2. Assay 2

**Figure 2:**
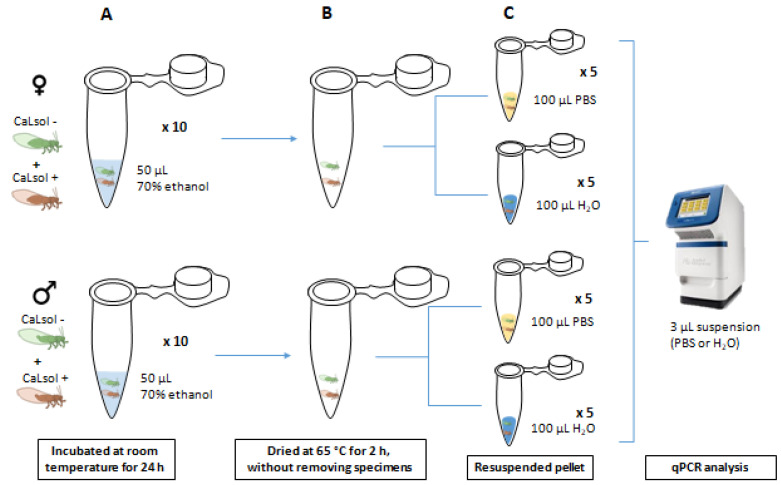
Cross-contamination study: Assay 2. Couples of same sex psyllids, one CaLsol-positive and one CaLsol-free, were incubated in ethanol (**A**). Ethanol was dried without removing the specimens (**B**). The samples were resuspended in water or PBS before direct qPCR analysis (**C**). ♀: female; ♂: male.

Twenty same-sex couples of *B. trigonica*, one CaLsol-positive and one CaLsol-free, were introduced in a microtube with 50 μL of 70% ethanol (10 tubes with two males and 10 tubes with two females), and incubated at room temperature for 24 h (Figure 2). The tubes were dried at 65 °C for 2 h without removing specimens. Half of the tubes were resuspended in 100 μL of RNAse-DNAse free H_2_O and the other half in 100 μL of PBS. Volumes of 3 μL from H_2_O and PBS solutions were used as a template for qPCR following Lso protocol [51].

#### 2.8.3. Assay 3

**Figure 3:**
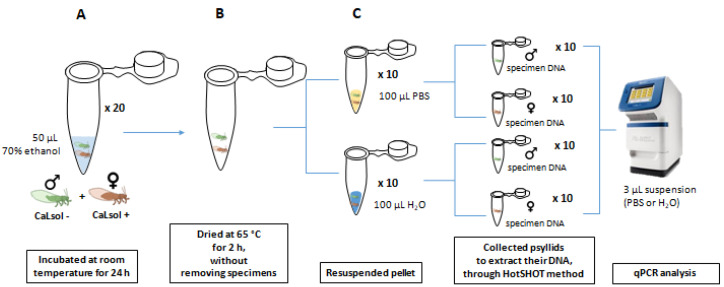
Cross-contamination study: Assay 3. Samples containing a pair of psyllids [one CaLsol-positive female (♀) and one CaLsol-free male (♂)] were incubated in ethanol (**A**). The ethanol was dried without removing the specimens (**B**). The samples were resuspended in water or PBS (**C**) and the psyllids were individually separated in different tubes before DNA extraction by the HotSHOT method.

Twenty couples of *B. trigonica*, one adult male CaLsol-free and one adult female CaLsol-positive, were introduced in 50 μL of 70% ethanol and incubated 24 h at room temperature (Figure 3). Tubes were dried at 65 °C for 2 h without removing specimens. Then, half of the tubes were resuspended in 100 μL of RNAse-DNAse free H_2_O and the other half in 100 μL of PBS. To detect whether there was cross-contamination from the positive female to the negative male insect, DNA extraction was performed from each specimen individually using the HotSHOT method.

### 2.9. Data Analysis

Statistical analysis was performed to elucidate significant differences among DNA extraction methods and specimen preparations according to the means of absorbance ratio value (A_260_/A_280_), DNA yield ratio (ng·mg^−1^) and cycle quantification (C_q_) values obtained with the two protocols previously mentioned. The comparison among the specimen preparation procedures (grinding, whole, cut-off head, and punctured abdomen) were conducted with the following methods: Chelex, HotSHOT, PBS, and DNeasy^®^; whereas the comparison among all the DNA extraction methods was performed using ground specimen preparation. Results of qPCR of samples stored 130 weeks, where compared with previous C_q_ values obtained. SPSS Statistic version 22 software (IBM) was used for statistical analysis. All the data recorded were checked for normality using Shapiro–Wilk W test. Comparisons between treatments were made by ANOVA–Welch (Gaussian variables) or by Kruskal–Wallis test (for non-Gaussian variables). Post hoc testing of Gaussian variables was carried out using the Games–Howell procedure. *p*-values lower than 0.05 were considered statistically significant.

## 3. Results

### 3.1. DNA Yield and Purity

Results of DNA yield (ng·mg^−1^) and purity (A_260_/A_280_) of the seven DNA extraction methods and their comparison by using ground specimen preparation are shown in Table 2. Yield ratio of DNA ranged between 1757.1 ± 295.8 ng·mg^−1^ and 24,328 ± 1638 ng·mg^−1^ (mean ± SE) with significant differences among the methods evaluated (F = 43.438, df = 6, *p* < 0.000). Chelex, TRIsure™, and HotSHOT showed the highest values in DNA yield ratio followed by squashed on membrane, PBS, DNeasy^®^, and CTAB. Significant differences were also observed between methods when purity ratio was assessed (F = 51.530, df = 6, *p* < 0.000), where CTAB, DNeasy^®^ and TRIsure^TM^ provided the highest values. By contrast, PBS and squashed on membrane provided the lowest DNA purities.

Table 3 summarizes the comparison among the specimen preparations (grinding, whole, cut-off head, and punctured abdomen) according to their DNA yield. Chelex and HotSHOT provided the highest values in DNA yield with no significant differences among preparation procedures. However, significant differences in DNA yield within specimen preparation were observed with PBS (F = 18.741, df = 3, *p* = 0.007) and DNeasy (F = 2.96, df = 3, *p* = 0.041). Ground preparation and the punctured abdomen gave the highest NanoDrop concentration readings in PBS and DNeasy^®^, respectively, while the intact full insect preparation obtained the lowest values.

Comparison among specimen preparations and descriptive parameters in DNA purity (A_260_/A_280_) are summarized in Table 4. DNeasy^®^ provided the best values in DNA purity, without significant differences among specimen preparation; meanwhile, all values for Chelex, HotSHOT, and PBS were below the range usually considered acceptable for molecular purposes.

### 3.2. Conventional PCR and qPCR

**Figure 4:**
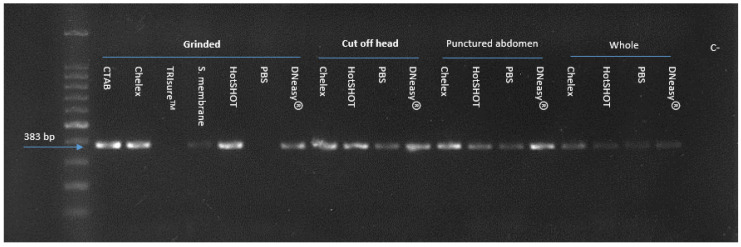
Agarose gel electrophoresis of products by Lso TX 16/23 primers, showing the 383 bp CaLsol-specific DNA fragment (arrow), obtained for seven DNA extraction methods and four specimen preparations. C-: negative control (right line). A 100 bp DNA ladder was included (left line).

Results of conventional PCR for the detection of CaLsol are shown in Figure 4. Positive PCR results were consistently obtained with DNA extracted from all the non-destructive preparations: cut-off head, punctured abdomen, and whole. No PCR products were generated with DNAs from ground psyllids samples extracted with TRIsure™ and PBS. The brightest bands were observed in all specimen preparations in Chelex method, while whole specimen preparation in PBS, DNeasy, and HotSHOT provided the lowest intensity bands.

Table 5 shows the results of the CaLsol detection of each DNA extraction method by using two different qPCR amplification protocols in ground samples. Data are expressed as positive result proportion (PP) and cycle quantification values (C_q_). In general, the Lso protocol turned out to be more sensitive than CaLsol protocol to detect the bacteria from psyllid tissues (Z = −5.672; *p* < 0.000). According to the PP, CaLsol was detected in all samples regardless of the extraction method and qPCR protocol, with the exception of TRIsure^TM^ and PBS. Significant differences in C_q_ values of the DNA extraction methods were obtained from both, CaLsol (F = 71.347, df = 6, *p* < 0.000) and Lso (F = 76.347, df = 6, *p* < 0.000) protocols. By means of CaLsol protocol, the HotSHOT method showed the lowest C_q_ values (mean ± SE, 18.15 ± 0.36) followed by Chelex, DNeasy^®^, and CTAB without significant differences. Similarly, with Lso protocol, the HotSHOT method showed the lowest C_q_ values (mean ± SE, 16.64 ± 0.23) without significant differences with Chelex and DNeasy^®^. The lowest sensitivity was obtained in both qPCR protocols with TRIsure^TM^ (mean ± SE, CaLsol protocol: 29.80 ± 0.70; Lso protocol: 27.30 ± 0.88) and squashed on membrane (mean ± SE, CaLsol protocol: 29.85 ± 0.79; Lso protocol: 24.80 ± 1.03).

Table 6 shows results of qPCR analysis by Li et al. [51] and Teresani et al. [7] protocols in four specimen preparations compared with the different DNA extraction methods evaluated. The Lso protocol showed a higher sensitivity compared to the CaLsol protocol as demonstrated by the lower C_q_ values obtained for all the samples analyzed regardless of the DNA extraction method used. In both protocols, the HotSHOT provided the lowest C_q_ values. Ground specimens showed the best results among other preparation methods except for the PBS procedure in CaLsol protocol. The combination of HotSHOT DNA extraction method and ground preparations provided the highest sensitivity. When Chelex was used, significant differences were shown between ground and non-destructive preparation procedures in CaLsol protocols (F = 17.274, df = 3. *p* = 0.001). Significant differences were also shown among specimen preparations when Lso protocol and DNeasy^®^ extraction were used (F = 31.355, df = 3, *p* < 0.000). However, no differences were observed among preparations in PBS, HotSHOT, and DNeasy^®^ with CaLsol protocol; or PBS and HotSHOT, with Lso protocol.

To check the usability of the isolated DNA after a long-term storage, extraction methods of ground specimen preparation with 100% of positive result proportion by qPCR were newly evaluated after 130 weeks of storage (Table 7). It was possible to detect CaLsol in all DNAs samples extracted by DNeasy^®^, Chelex, and CTAB by both qPCR protocols used. However, an increase in C_q_ values was observed after storage by CaLsol protocol (ranging from 1.8 to 5.7) and higher by Lso protocol (ranging from 4 to 7.9). In both qPCR protocols, DNeasy^®^ and Chelex showed the lowest differences in C_q_ values when pre- and post-DNA storage were compared.

### 3.3. Morphological State of Psyllids after DNA Extraction

**Figure 5:**
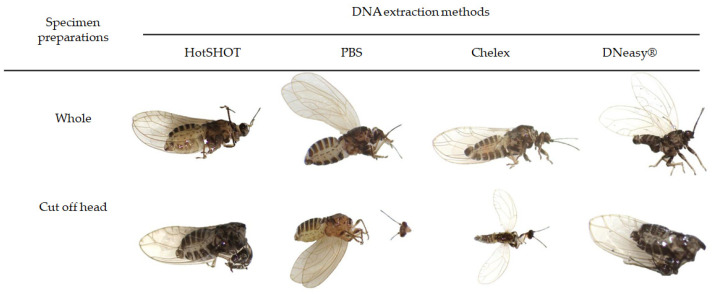
‘Whole’ and ‘Cut off head’ individuals after DNA extraction with HotSHOT, PBS, Chelex, and DNeasy^®^.

After DNA extraction, all non-destructive specimen preparations tested (whole specimen, cut off head, and punctured abdomen) perfectly allowed a morphological identification of the psyllid to specific level (Figure 5). Under the conditions laid down in this work [49], DNA extraction with DNeasy^®^ induced fragile structures in the psyllid that were easily broken when touched. This was inconvenient when handling the insect during preparation for its subsequent identification.

### 3.4. Time Consumed, Cost Estimation, and Residues Generated

Table 8 summarizes the time consumed, cost of consumables, and reagent residues of each DNA extraction method evaluated.

Considering the time consumed during handling, the fastest methods of DNA extraction were squashed on membrane (15 min), followed by HotSHOT (30 min), and PBS (35 min). TRIsure^TM^ (2 h) and CTAB (2 h 20 min), both including an extra period of 2 h for sample drying, required longer times to complete the procedure. Chelex and DNeasy^®^ required an extra incubation time period of 24 h, although they only took 30 min of effective handling of the samples. Regarding chemical residues produced by the different protocols, Chelex, squashed on membrane, HotSHOT, and PBS did not generate waste, unlike DNeasy^®^, TRIsure ^TM^, and CTAB. In addition, these last two extraction methods produced hazardous residues such as CTAB buffer, chloroform: isoamyl alcohol (24:1), chloroform, and TRIsure^TM^ lysis buffer.

The estimated cost of consumables per sample of CTAB, HotSHOT, Chelex, and PBS (ranging between 0.07 and 0.33 € per sample) was considerably lower than the cost of the commercial kits DNeasy^®^ or TRIsure^TM^ (2.56 € and 1.16 € per sample, respectively).

### 3.5. Cross Contamination Assay

The results of the cross-contamination assays during psyllid handling are shown in Table 9. The analysis of ethanol samples in which CaLsol-positive *B. trigonica* were incubated (assay 1) resulted in clear positive by qPCR from both PBS and water suspensions, with C_q_ values (mean ± SE) of 29.7 ± 0.6 and 30.4 ± 3.6, respectively. In the mixture of ethanol with CaLsol-positive psyllid and ethanol with CaLsol-negative psyllid, the bacterium was only detected in those samples resuspended in PBS (29.0 ± 0.6). When CaLsol-positive and CaLsol-free *B. trigonica* of the same sex were co-incubated (assay 2), the target was detected in water suspensions (31.8 ± 1.0) and PBS (28.9 ± 0.4). In both assays, the qPCR reactions ran with samples resuspended in PBS provided lower C_q_ values than those resuspended in water. The males of *B. trigonica* CaLsol-free co-incubated with CaLsol positive females (assay 3) gave positive results in all cases when they were individually analyzed by qPCR. Values of C_q_ for the male specimens were considerably higher (33.5 ± 0.4 in water and 30.9 ± 2.3 in PBS) than those obtained with the females (17.3 ± 0.8 in water and 20.2 ± 2.5 in PBS).

## 4. Discussion

In vector-borne diseases, such as those caused by Liberibacters that are transmitted by psyllids, it is usually necessary to detect the pathogen in the vector by molecular techniques such as PCR that require DNA extraction. Moreover, pathogen detection is sometimes a preliminary step before vector species identification, so keeping the insect structure is required. Moreover, a good DNA extraction method should be simple and fast to perform, as well as efficient in order to obtain sufficient DNA at a reasonable purity level. Comparison studies of different methods to extract DNA from insects have been carried out in previous works [41,48,50,59]. They highlight the importance of developing an accurate, fast, simple, cheap, and environmentally friendly method to facilitate and standardize work in laboratories.

This study evaluates four specimen preparations and seven DNA extraction methods, including commercial kits and in-house protocols, to detect CaLsol in the psyllid vector *B. trigonica*. The main goal was to find a non-destructive method to detect this endogenous bacterium by PCR, allowing for the subsequent morphological identification of the psyllid. For each method, time consumed, monetary cost, and hazardous residues generated were determined. Cross-contamination with CaLsol was also assessed between psyllids handled in vials with ethanol as preservative solution.

Contrary to what might be initially expected, the crushing of the insects prior to DNA extraction did not result in a considerable increase of the total DNA yield when compared to non-destructive preparation methods. However, our study suggests that crushing specimens may improve the qPCR sensitivity for detecting CaLsol. In some ground samples, negative results were obtained by conventional PCR, which might indicate the presence of inhibitors that interfere in the reaction. In addition, our data confirm that an accurate qualitative detection of the target pathogen is also possible with four non-destructive methods, thereby allowing the subsequent morphological identification of the insects. The idea that destructive procedures are necessary to release the bacteria from the internal insect tissues, improving the detection of the target, is not fulfilled here. Obtaining enough DNA from a small insect such as *B. trigonica* (approximately 4 mm in length) is already a difficult task [60,61], and it is even more difficult to detect an endogenous bacterium that may be present in a very low concentration. However, the qPCR system provides sufficient sensitivity and specificity to allow for the detection of a few target molecules in an almost intact insect sample. In our case, CaLsol was detected in less than 0.05 µg of insect, and that level of detection was achieved through non-destructive preparations methods (unprocessed specimens, cut off head, or punctured abdomen).

DNA yield and purity were unrelated to the sample preparation procedure, but mainly to the DNA extraction protocol used. The nucleic acid purification methods showed differences among yield and purity in the DNA obtained. The best results were achieved by Chelex and TRIsure^TM^ that provided higher values in terms of DNA yield compared to the other methods. However, DNA purity obtained by these two methods was poor with an absorbance ratio (A_260_/A_280_) below 1.8, which is considered the minimum accepted value when determining the quality of DNA [62]. These observations could indicate the presence of impurities not removed during DNA extraction, which can lead to erroneous DNA concentration readings, obtaining abnormally high values. The Chelex method involves the addition of a chelating ion exchange resin and proteinase K that allow DNA release after a heating step [53]. Part of these products remains in the sample after DNA extraction as impurities, which may interfere with the correct determination of yield of the nucleic acids. However, it seems that there are not PCR inhibitors affecting the efficiency of the amplification by conventional PCR or qPCR. Chelex also allows valid amplifications for sequencing, genotyping, specific detection, or other applications and it is largely used to extract DNA from different matrix such as forensic materials, insects, plants, bacteria, etc. [63,64,65,66]. TRIsure^TM^, which was developed to isolate both RNA and DNA [57], demonstrated poor sensitivity to detect CaLsol by PCR or qPCR. This low efficiency during the amplification could be caused by traces of chloroform or/and ethanol, which might remain in the extracted DNA, inhibiting the PCR reaction. The HotSHOT method rendered intermediate values in the DNA yield with poor quality, although, data from conventional PCR and qPCR revealed that it was one of the most sensitive in detecting CaLsol from psyllids.

The CTAB and the DNeasy^®^ methods, widely used as DNA extraction procedures in diagnostic laboratories [13], provided A_260_/A_280_ between 1.8 and 2.0, indicating high purity of the DNA [67], but with low DNA yields comparable to those obtained by PBS and squashed on membrane methods. Similar results were achieved for DNeasy^®^ in western corn rootworm beetles [41] and in mealybugs [59], with a low DNA yield but high purity. Finally, the low-quality of the DNA obtained with PBS and squashed on membrane was not consistently and efficiently amplified by conventional PCR or qPCR. In both methods, the DNA was probably accompanied by PCR inhibitors causing a loss of efficiency or even in false negative results.

The analysis of samples which had been stored for 130 weeks at −20 °C showed a decrease in sensitivity for CaLsol detection by qPCR. Cycle quantification values were higher than those obtained previously, demonstrating a possible partial degradation of DNA, as it was noted in previous research [67,68,69]. However, the DNA extraction methods assessed in this study allowed the use of the isolated DNAs for more than 2 years with ground sample preparation for detection purposes. According to Hajibabbaei et al. [60] the storage potential of isolated DNA obtained through DNeasy^®^ is moderate, being feasible one year at −20 °C, and similar results were obtained by Chakraborty et al. [69] in which DNA extracted with CTAB was stable, with a durability of about two years.

In terms of time consumption, TRIsure^TM^ and CTAB were the most laborious methods, involving several steps during sample processing. Chelex and DNeasy^®^ are two simple and easy to perform extraction procedures, but they include an overnight incubation step that extends the time to obtain the isolated DNA [49,53]. For DNeasy^®^, we followed the method optimized by Sjölund [49] for the detection of CaLsol in psyllids, although other studies have used this kit according to the manufacturer’s recommendation or modifying the procedure by reducing the incubation periods [41,59,70]. Squashed on membrane and HotSHOT were the fastest procedures and excellent alternatives when a quick screening is required.

Comparing the cost of all the extraction methods, taking as a reference the cheapest ones (HotSHOT and PBS, 0.07 €/sample), DNeasy^®^, TRIsure^TM^, CTAB, Chelex, and squashed on membrane were approximately 36, 17, 5, 4, and 3 times more expensive, respectively. At the same time, extracting DNA with a minimum of hazardous residues is always advisable and a factor to be taken into account when choosing the most appropriate procedure. TRIsure^TM^ and CTAB generated hazardous reagents [57,71], which required the use of proper facilities—such as fume hoods, personal protective equipment—and a protocol to manage them safely, according to the European legislation (Regulation (CE) 1272/2008). Moreover, an additional disadvantage of using TRIsure^TM^ is the risk of burns in contact with the skin, mucous membranes, or eyes [57]. Other DNA extraction procedures such as DNeasy^®^ or CTAB require the use of storage containers and the costly provision of waste collection and treatment services. Therefore, the DNA extraction methods assessed for the detection of CaLsol in *B. trigonica*, offers a wide range of options regarding time consumption, price, and residues handling, thereby making it a process adaptable to the needs and circumstances of each laboratory.

In summary, HotSHOT and squashed on membrane were not only the fastest methods, but also the cheapest. Several other authors have also defined squashed on membrane and HotSHOT as fast methods to obtain DNA for diagnosis purposes [7,55,59]. Truett et al. [56] indicated that HotSHOT produced less nonspecific amplification than traditional methods and provided the cleanest PCR products. Results obtained in this work revealed that HotSHOT is the most sensitive method for detecting CaLsol by qPCR in psyllid samples. In addition, the simplicity of this method makes the procedure highly recommended for the analysis of psyllid samples in the detection of CaLsol, because it enables the performance in a single tube, keeps the structure of the specimen intact, requires less than 30 min, and can be carried out without generating contaminating residues.

During the development of this study, it was possible to detect CaLsol in psyllid samples without specimen preparation using the PBS method. This observation might indicate that, once collected, the insect could release the endogenous CaLsol into the surrounding environment. This is especially important during field prospections since many insects are usually collected and mixed in the same vials containing ethanol or other preservative solutions. Our results demonstrate that CaLsol-positive psyllids were able to cross-contaminate CaLsol-negative psyllids collected in the same tubes, resulting in positive reactions by qPCR from specimens not initially carrying the bacteria. These results must be considered in studies which seek to determine the prevalence of this endogenous organism in order to avoid an overestimation of its proportion in the psyllid population. Thus, it is advisable to collect the insects with CO_2_ or by freezing at −20 °C, and to store the specimens in individual vials with the desired ethanol solution.

International trade of plants, vegetables, and fruits between different countries has caused the worldwide spread of harmful pests. The correct taxonomic identification of pests is crucial in order to adopt the most appropriate and effective control measures. Nowadays, the use of barcoding molecular techniques for this aim is more frequent and, consequently, the available information of specimen’s sequences in databases is increasing [72]. When recognition and identification of arthropods by morphological approaches is required, keeping the arthropod exoskeleton after DNA extraction is mandatory for proper identification by trained specialists. Our work, which improves the knowledge on different specimen preparations and DNA extraction methods, was aimed at finding the best method to detect CaLsol in the vector. However, it is suggested that it could also be useful for the identification of insects using molecular barcoding techniques.

Moreover, information shown in this work could be a valuable guide for the detection of other species of these bacteria in their vectors, with a special focus on the species ‘*Ca.* L. africanus’ (CaLaf), ‘*Ca.* L. americanus’, and ‘*Ca.* L. asiaticus’ (CaLas); associated with Huanglongbing (HLB), the most severe disease of citrus plants (*Citrus* spp.) [1,73]; and transmitted by *Diaphorina citri* [6,73,74] and *Trioza erytreae* [6,75]. These vectors could be handled as *B. trigonica* in this study as an alternative to the current protocols used [76,77]. This work provides a wide range of options to detect CaLsol in its vectors according to the purpose of the study. Among the extraction methods evaluated, HotSHOT was the fastest, cheapest, safest, and did not require destructive preparation to consistently detect CaLsol in psyllid vectors by qPCR.

## Figures and Tables

**Table 1:** Number and sex of *B. trigonica* specimens used to evaluate each DNA extraction method and preparation procedure.

	Specimen Preparation			
Methods *	Whole	Grinding	Cut-Off Head	Punctured Abdomen
CTAB	N/A	7 ♀ + 7 ♂	N/A	N/A
Chelex	7 ♀ + 7 ♂	7 ♀ + 7 ♂	7 ♀ + 7 ♂	7 ♀ + 7 ♂
TRIsure^TM^	N/A	7 ♀ + 7 ♂	N/A	N/A
Squashed on membrane	N/A	7 ♀ + 7 ♂	N/A	N/A
HotSHOT	7 ♀ + 7 ♂	7 ♀ + 7 ♂	7 ♀ + 7 ♂	7 ♀ + 7 ♂
PBS	7 ♀ + 7 ♂	7 ♀ + 7 ♂	7 ♀ + 7 ♂	7 ♀ + 7 ♂
DNeasy^®^	7 ♀ + 7 ♂	7 ♀ + 7 ♂	7 ♀ + 7 ♂	7 ♀ + 7 ♂

* Methods: CTAB [52]; Chelex 100 (Biorad) [53]; TRIsure^TM^; Squashed on membrane [54]; HotSHOT [55,56]; PBS, saline phosphate buffer + Tween 20 (5%); and DNeasy^®^ Blood and Tissue kit (Qiagen) [49]. N/A: Not applicable. ♀: female; ♂: male.

**Table 2:** Comparison among DNA yield ratios and DNA purity ratios (mean ± SE, *n* = 14) of seven DNA extraction methods with ground specimen preparation.

Methods	DNA Yield (ng·mg^−1^) *	DNA Purity (A_260_/A_280_)
**CTAB**	1757 ± 296 **d**	1.89 ± 0.11 **a**
**Chelex**	24,328 ± 1638 **a**	1.15 ± 0.03 **b**
**TRIsure^TM^**	20,746 ± 4511 **ab**	1.59 ± 0.07 **a**
**Squashed on membrane**	6471 ± 713 **cd**	0.67 ± 0.05 **c**
**HotSHOT**	11,964 ± 1187 **bc**	1.03 ± 0.04 **b**
**PBS**	3850 ± 489 **cd**	0.62 ± 0.06 **c**
**DNeasy^®^**	3271 ± 380 **d**	1.85 ± 0.07 **a**

* DNA yield was calculated according to Chen et al. [41] based on DNA volume and average adult body weight of *B. trigonica*. Data and statistical analyses are presented in columns. The different letters mean significant differences among DNA extraction methods (*p* < 0.05).

**Table 3:** Comparison of DNA yield ratios * expressed in ng·mg^−1^ (mean ± SE, *n* = 14) of the specimen preparation procedures in four DNA extraction methods.

Specimen Preparations	DNA Extraction Methods			
	Chelex	HotSHOT	PBS	DNeasy^®^
**Whole**	31,164 ± 3261 **a**	8078 ± 1271 **a**	1582 ± 211 **b**	2386 ± 256 **b**
**Grinding**	24,328 ± 1638 **a**	11,964 ± 1187 **a**	3850 ± 489 **a**	3271 ± 380 **ab**
**Cut off head**	32,936 ± 2816 **a**	8664 ± 1021 **a**	2750 ± 388 **ab**	3121 ± 320 **ab**
**Punctured abdomen**	31,814 ± 1956 **a**	10,643 ± 1523 **a**	2661 ± 357 **ab**	3786 ± 373 **a**

* DNA yield ratio calculated according to Chen et al. [41] based on DNA volume and average adult body weight of *B. trigonica*. Statistical analysis is shown in columns. Data followed by different letters mean significant differences among specimen preparations (*p* < 0.05) within the same method.

**Table 4:** Comparison among DNA purity ratios A_260_/A_280_ (mean ± SE, *n* = 14) of the specimen preparation procedures in four DNA extraction methods.

Specimen Preparations	DNA Extraction Methods			
	Chelex	HotSHOT	PBS	DNeasy^®^
**Whole**	1.17 ± 0.02 **a**	0.80 ± 0.06 **b**	1.38 ± 0.16 **a**	1.62 ± 0.09 **a**
**Grinding**	1.15 ± 0.03 **a**	1.03 ± 0.04 **a**	0.62 ± 0.06 **b**	1.85 ± 0.07 **a**
**Cut off head**	1.19 ± 0.02 **a**	0.98 ± 0.04 **ab**	1.15 ± 0.07 **a**	1.78 ± 0.06 **a**
**Punctured abdomen**	1.10 ± 0.03 **a**	0.79 ± 0.06 **b**	1.23 ± 0.11 **a**	1.85 ± 0.07 **a**

Statistical analysis is shown in columns. Data followed by different letters mean significant differences among specimen preparations (*p* < 0.05) within the same method.

**Table 5:** CaLsol detection in ground specimen preparation with seven DNA extraction methods by two qPCR protocols.

DNA Extraction Methods	qPCR Protocols *			
	CaLsol		Lso	
	PP	Cq	PP	Cq
**CTAB**	100	21.9 ± 1.0 **ab**	100	22.4 ± 1.1 **c**
**Chelex**	100	20.1 ± 0.5 **ab**	100	18.7 ± 0.4 **ab**
**TRIsure^TM^**	100	29.8 ± 0.7 **c**	92.8	27.3 ± 0.9 **c**
**Squashed on membrane**	100	29.8 ± 0.8 **c**	100	24.8 ± 1.0 **c**
**HotSHOT**	100	18.1 ± 0.4 **a**	100	16.6 ± 0.2 **a**
**PBS**	64.3	27.5 ± 1.2 **bc**	64.3	21.6 ± 0.7 **bc**
**DNeasy^®^**	100	20.2 ± 0.2 **ab**	100	18.1 ± 0.2 **ab**

* qPCR protocols: CaLsol [7] and Lso [51]. Results are expressed as positive results proportion. PP = 100·(No. of samples CaLso positives)/(no. of samples analyzed) and C_q_ mean ± SE (*n* = 14). Data and statistical analysis are shown in columns. Different letters mean significant differences among DNA extraction methods (*p* < 0.05).

**Table 6:** CaLsol detection analysis in four specimen preparations and four DNA extraction methods by two qPCR protocols.

qPCR Protocols *	DNA Extraction Methods	Specimen Preparation			
		Whole	Ground	Cut-Off Head	Punctured Abdomen
**CaLsol**	**Chelex**	23.4 ± 0.8 **b**	20.1 ± 0.5 **a**	23.5 ± 1.1 **b**	23.9 ± 0.7 **b**
	**HotSHOT**	18.9 ± 0.7 **a**	18.1 ± 0.4 **a**	18.6 ± 0.6 **a**	20.1 ± 0.8 **a**
	**PBS**	27.4 ± 1.4 **a**	27.5 ± 1.2 **a**	26.3 ± 0.7 **a**	28.1 ± 1.1 **a**
	**DNeasy^®^**	25.4 ± 1.1 **a**	20.2 ± 0.2 **a**	22.4 ± 0.7 **a**	23.6 ± 1.0 **a**
**Lso**	**Chelex**	21.9 ± 0.7 **b**	18.7 ± 0.3 **a**	21.9 ± 0.9 **b**	21.6 ± 0.6 **b**
	**HotSHOT**	18.0 ± 0.5 **a**	16.6 ± 0.2 **a**	17.8 ± 0.5 **a**	18.4 ± 0.8 **a**
	**PBS**	23.9 ± 1.5 **a**	21.6 ± 0.7 **a**	21.7 ± 0.8 **a**	24.0 ± 1.2 **a**
	**DNeasy^®^**	25.1 ± 0.7 **c**	18.1 ± 0.1 **a**	20.9 ± 0.9 **ab**	21.5 ± 0.9 **bc**

* qPCR protocols: CaLsol [7] and Lso [51]. Results are expressed as C_q_ mean ± SE (*n* = 14). Statistical analysis is shown in rows. Data followed by different letters mean significant differences among specimen preparations (*p* < 0.05).

**Table 7:** Comparison of qPCR results by CaLsol and Lso protocols before (t = 0) and after 130-weeks of storage (t = 130) of ground samples in each method.

DNA Extraction Methods	qPCR Protocols *					
	CaLsol			Lso		
	PP	Cq (t = 0)	Cq (t = 130)	PP	Cq (t = 0)	Cq (t = 130)
**CTAB**	100	22.5 ± 1.7	28.2 ± 1.3	100	22.5 ± 1.3	27.4 ± 1.2
**Chelex**	100	21.0 ± 1.2	23.6 ± 0.6	100	19.4 ± 0.6	23.5 ± 0.6
**Squashed on membrane**	66.7	29.7 ± 1.3	32.4 ± 1.7	100	25.2 ± 1.5	31.2 ± 1.1
**HotSHOT**	100	18.0 ± 0.8	22.8 ± 0.4	83.3	17.4 ± 0.7	25.3 ± 1.5
**DNeasy^®^**	100	23.6 ± 2.4	25.4 ± 3.4	100	20.3 ± 1.9	24.3 ± 2.0

* qPCR protocols: CaLsol [7] and Lso [51]. Results are expressed as positive result proportion after 130 weeks of storage, PP = 100·(no. of samples CaLso positives)/(no. of samples analyzed) and C_q_ mean ± SE (*n* = 10).

**Table 8:** Summary of protocol according of time consumed, cost per sample, and reagent residues generated for each of the evaluated DNA extraction method.

DNA Extraction Methods	Time Consumed *	Cost Consumables (€/Sample)	Hazardous Reagent Residues *	Non-Hazardous Reagent Residues *
**CTAB**	2 h 20 min **	0.33	4 mL buffer CTAB + 4 mL chloroform:isoamyl alcohol (24:1)	1.2 mL isopropanol+ 10 mL 70% ethanol
**Chelex**	30 min ***	0.26	-	-
**TRIsure^TM^**	2 h **	1.16	10 mL TRIsure^TM^ + 2 mL chloroform	10 mL 0.1 M sodium citrate with 10% ethanol + 3.5 mL 100% ethanol + 15 mL 75% ethanol
**Squashed on membrane**	15 min	0.24	-	-
**HotSHOT**	30 min	0.07	-	-
**PBS**	35 min	0.07	-	-
**DNeasy^®^**	30 min ***	2.56	-	0.2 mL proteinase K, 2 mL ethanol 100% and buffers: 1.8 mL ATL, 2 mL AL, 5 mL AW1 and 5 mL AW2.

* Estimated by processing 10 samples. ** It requires two extra hours of drying time (not included). *** It requires an overnight incubation period (not included).

**Table 9:** Detection of CaLsol by qPCR analysis according to Li et al. (2009) in cross-contamination assays.

Assay No.	Description	Sample Analyzed	PP **	C_q_, Mean ± SE, *n* = 10 ***
1	10 specimens CaLsol- (5♀ and 5♂) (A)	H_2_O	0%	nd
	10 specimens CaLsol+ (5♀ and 5♂) (B)	H_2_O	100%	30.4 ± 3.6
	10 mixtures of ethanol (A + B)	H_2_O	0%	nd
	10 specimens CaLsol- (5♀ and 5♂) (A)	PBS	0%	nd
	10 specimens CaLsol+ (5♀ and 5♂) (B)	PBS	100%	29.7 ± 0.6
	10 ethanol mixture (A + B)	PBS	100%	29.0 ± 0.6
2	10 same-sex couples (5 ♀ CaLsol+ ♀ CaLsol- and 5 ♂ CaLsol+ ♂ CaLsol−)	H_2_O	100%	31.8 ± 1.0
	10 same-sex couples (5 ♀ CaLsol+ ♀ CaLsol- and 5 ♂ CaLsol + ♂ CaLsol−)	PBS	100%	28.9 ± 0.4
3 *	10 ♂ specimens CaLsol-	Insect	100%	33.5 ± 0.4
	10 ♀ specimens CaLsol+	Insect	100%	17.2 ± 0.8
	10 ♂ specimens CaLsol-	Insect	100%	30.9 ± 2.3
	10 ♀ specimens CaLsol+	Insect	100%	20.2 ± 2.5

* The DNA analyzed was extracted from individual insects previously incubated in pairs (one positive female + one negative male). ** PP (Positive result proportion) = 100 (no. of samples CaLso positives)/ (no. of samples analyzed). *** Cycle quantification values of qPCR analysis by Lso protocol [51]. nd: not detected. ♀: female; ♂: male.

## Supplementary Materials

The following supporting information can be downloaded at: https://www.mdpi.com/article/10.3390/microorganisms10061104/s1, Table S1: References, sequences and amplification conditions of the PCR and qPCR protocols used in the present study.
