# Supplementary material for: Assessment of Psyllid Handling and DNA Extraction Methods in the Detection of ‘Candidatus Liberibacter Solanacearum’ by qPCR
Source: Microorganisms. 2022 May 26;10(6):1104. doi: 10.3390/microorganisms10061104 (PMC9230594; doi:10.3390/microorganisms10061104)
Supplement: Supplementary file 1 [file microorganisms-10-01104-s001.zip › microorganisms-1736145-supplementary.pdf]

Table S1. References, sequences and amplification conditions of the PCR and qPCR protocols used in the present study.

R *	PCR **	Protocol		Conditions ***		
		**Name**	**Sequences (5′-3′)**	**Cycles**	**T (°C)**	**Time**
[58]	C	LsoTX 16/23F	AATTTTAGCAAGTTCTAAGGG	1	98	30 s
				35	98	10 s
					62	20 s
		LsoTX 16/23R	GGTACCTCCCATATCGC		72	30 s
				1	72	7 min
[51]	Q	CaLsppF	GCAG GCCTAACACATGCAAGT	1	95	10
		CaLsppR	GCAC ACGTTTCCATGCGTTAT	45	95	15 s
		CaLsolP	FAM-AGCGCTTATTTTTAATAGGAGCGGCAGACG-TAMRA		60	60s
[7]	Q	LsoF	GTC GAG CGC TTA TTTTTA ATA GGA	1	95	20 s
		HLBr	GCGTTATCCCGTAGAAAAAGGTAG	40	95	1s
		HLBp	FAM-AGACGGGTGAGTAACGCG-BHQ-1		58	40s

* R: References.

** C: conventional PCR protocol; Q: quantitative PCR protocol.

*** Amplification conditions by conventional PCR and qPCR.
